# Supplementary material for: Complexome profiling of the Chlamydomonas psb28 mutant reveals TEF5 as an early PSII assembly factor
Source: Plant Cell. 2025 Mar 17;37(6):koaf055. doi: 10.1093/plcell/koaf055 (PMC12164590; doi:10.1093/plcell/koaf055)
Supplement: koaf055_Supplementary_Data [file koaf055_supplementary_data.zip › psb28_interactive.zip!/plots/intensity/qualityPlots/peptides/FKB16-8_Cre02.g095122.t1.1.html]

### Cre02.g095122.t1.1

Trivial name: FKB16-8  
SeqCoverage [median]: 25.30  
Mass: 20.87 kDa  
  
 

| Sequence | MissedCleavage | Mass [Da] | wtIntensity | muIntensity | Score |
| --- | --- | --- | --- | --- | --- |
| AFEIAVSNMK | 0 | 1108.56 | 0.00 | 1539.44 | 120.15 |
| FTGELISQDLK | 0 | 1249.66 | 783.92 | 1992.53 | 180.09 |
| IDNTSVRDEPYEFK | 1 | 1711.81 | 0.00 | 585.74 | 38.19 |
| YGPQPAELGGQR | 0 | 1271.63 | 0.00 | 1701.58 | 71.45 |
|  |  |  | 783.9 | 5819.3 |  |
